# Endodontic Management of Mandibular Second Molar Fused to a Supernumerary Tooth, Using Spiral Computed Tomography as a Diagnostic Aid: A Case Report

**DOI:** 10.1155/2012/614129

**Published:** 2012-07-31

**Authors:** Kavitarani Rudagi, B. M. Rudagi, Sandeep Metgud, Rahul Wagle

**Affiliations:** ^1^Department of Conservative Dentistry and Endodontics, Sharad Pawar Dental College, Sawangi (Meghe), Maharashtra, Wardha, India; ^2^Department of Oral and Maxillofacial Surgery, Sharad Pawar Dental College, Sawangi (Meghe), Wardha, India; ^3^Department of Conservative Dentistry and Endodontics, Rural Dental College, Pravara Institute of Medical Sciences, Loni, India

## Abstract

Fusion is a developmental anomaly characterized by the union of two adjacent teeth. In this paper we report a rare case of fusion involving permanent mandibular second molar with supernumerary tooth. The rarity with which this entity appears, along with its complex characteristics, often makes it difficult to treat. The use of high-end diagnostic imaging modalities such as spiral computed tomography can help the clinician in making a confirmatory diagnosis and determining the treatment plan before undertaking the actual treatment. In the present case, we have used spiral computed tomography (SCT) for better understanding the complicated root canal morphology of the fused tooth and successful management of this rare case.

## 1. Introduction

Teeth with morphoanatomic changes pose a challenge in endodontic therapy. It is imperative that aberrant anatomy is identified before and during root canal treatment of such teeth. Morphoanatomic changes in teeth may be divided according to the site of their occurrence, that is, tooth crown, roots, and root canals. Fusion and gemination are irregularities in tooth development [1]. Pindborg defined fusion as the union between dentin and/or enamel of two or more separate developing teeth [2]. The incidence of fusion is <1% in the Caucasian population [3]. Clinically, it is often difficult to differentiate between fusion and gemination and it is common to refer these anomalies as “double teeth” [4, 5].

The degree of fusion depends on the stage of tooth development that has occurred at the time of fusion, with the union of dentin being the main criterion. Fused teeth may contain separate pulp canals or share a common pulp canal. Fusion may occur between two normal teeth or between a normal tooth and a supernumerary tooth. Supernumerary tooth is an additional entity to the normal series and is seen in all the quadrants of the jaw [6]. When the supernumerary tooth occurs distal to the molar, it is called a distomolar, and when it erupts buccal or lingual to the molar, it is called a paramolar. Supernumerary teeth might occur singly or might be fused to their normal counterpart [7]. The occurrence of fusion in permanent posterior teeth is rare.

When fusion occurs between a molar and a supernumerary tooth as a result of their abnormal morphology and excessive crown width, problems with crowding, alignment, and occlusal functions can occur [8]. These teeth also tend to be greatly predisposed to caries and periodontal disease [9].

Radiographic examination is an essential component in the management of endodontic problems. However, the conventional intraoral periapical views produce only a 2-dimensional image of a 3-dismensional object, resulting in the superimposition of structures. Conventional multidetector computed tomography (CT) imaging has been widely used in medicine since the 1970s and was introduced in the endodontic field in 1990 [7]. In these areas, spiral computed tomography (SCT) has proved to be a useful diagnostic tool [10, 11].

 This paper presents the endodontic management of a fused mandibular second molar with a supernumerary tooth with the use of SCT as a diagnostic aid.

## 2. Case Report

**Figure 1:**
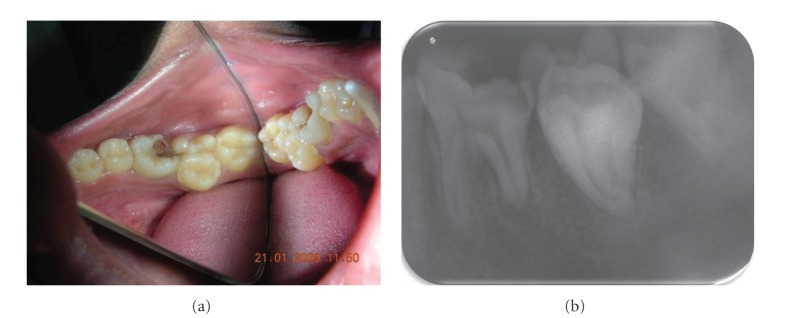
(a) Pre-operative photograph: Occlusal View. (b) Pre-operative radiograph revealing irregular morphology of tooth #20 & istal root resorption with #21.

A 20-year-old male patient presented to the department of conservative dentistry and endodontics with pain to hot and cold food in the left mandibular posterior region. On clinical examination, the mandibular first molar exhibited caries on the distoocclusal aspect and second molar exhibited abnormal crown morphology with 2 large extra cusps on the buccal aspect of the tooth (Figure 1(a)). Distinct developmental occlusogingival grooves between the supernumerary tooth and its normal counterpart were noticed. Despite the presence of the grooves, there was no discernible separation between the 2 teeth. Caries in the developmental groove on the mesial side of the tooth was evident.

**Figure 2:**
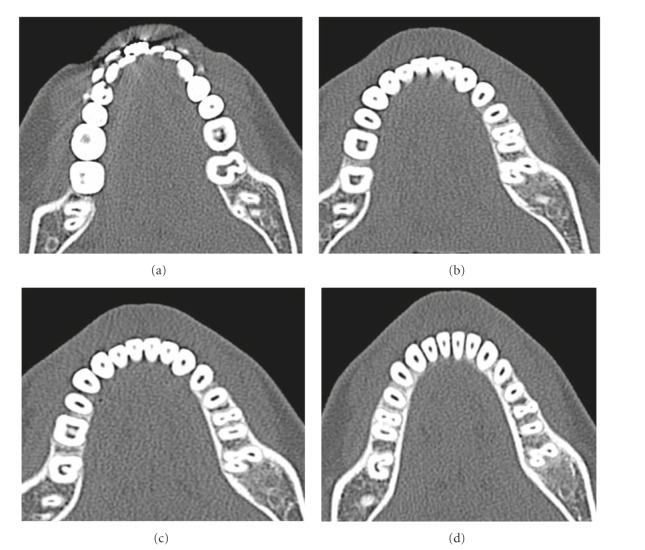
SCT Images of the Maxilla. (a) Axial view of the coronal third section of the roots of the left mandibular left second molar. (b) Axial view of the cervical third section of the roots of the mandibular left second molar. (c) Axial view of the middle third section of the roots of the left mandibular left second molar. (d) Axial view of the apical third section of the crown of the left mandibular left second molar.

 There was no previous history of trauma or any hereditary conditions. Medical history was noncontributory. All vital signs were found to be within normal limits. Oral examination revealed a normal set of dentition. The teeth 20 and 21 were not sensitive to palpation or percussion. The tooth responded to an electric pulp tester (Parkell Electronics, Farmingdale, NY, USA) and showed early response to the cold test (R C Ice, Prime Dental products, India). On radiographic examination, tooth 21 showed resorption with the distal root and the exact anatomy of tooth 20 that could not be clearly identified, but it was possible to recognize the complex structure of the pulp (Figure 1(b)). Therefore, to ascertain the complex root canal anatomy of the tooth in a 3-dimensional (3D) manner, dental imaging with the help of spiral computed tomography (SCT) was therefore planned. Informed consent from the patient was obtained, and a multislice SCT of the mandible was performed using the dental software Dentascan (GE Healthcare, Milwaukee, WI, USA). A 3D image of the mandible was obtained. The involved tooth was focused and the morphology was obtained in transverse, axial, and sagittal sections of 0.5 mm thickness (Figure 2).

The images revealed that the mandibular left second molar had 3 root canals and that there was connection between the paramolar and second molar in the pulpal chamber. The paramolar exhibited a type II canal (according to Wiene's classification).

## 3. Management

 Local anesthesia was administered and a rubber dam was applied. The prongs of the rubber dam retainer had to be trimmed to accommodate the unusual anatomy of the tooth. Endodontic access cavity was done on the occlusal surface using a no. 2 round bur and EX 24 bur (nonend cutting tapered fissure; Mani, Tochigi, Japan) (Figure 2(a)). Pulp extirpation was performed using a barbed broach (Denstply-Malliefer, Ballaigues, Switzerland) and K-files (Mani Inc, Tochigi, Japan). The canal was thoroughly debrided with a copious irrigation of sodium hypochlorite (2.5%), followed by saline (0.9%). Coronal flaring of the root canal was done using Gates-Glidden drills no. 1 to 2 (Mani Inc, Tochigi, Japan). The working length was determined using apex locator (Propex, DENTSPLY-Malleifer, Ballaigues, Switzerland) and confirmed radiographically (Figure 2(b)). Cleaning and shaping of the root canal system was completed using Protaper rotary system (Dentsply). Canals were copiously irrigated with sodium hypochlorite (2.5%), followed by saline. The canal was dried with sterile paper points and calcium hydroxide (Ultracal XS, Ultradent, South Jordan, UT, USA) was placed in the root canal and the access cavity was temporized with Cavit G (3 M ESPE, Germany). The patient was recalled after 1 week for obturation.

 After a week, the tooth was asymptomatic and the root canal was obturated using thermoplastic obturation technique (E&Q plus Meta Biomed Co ltd, Korea) and AH PLUS as a sealer. The access cavity was then sealed with resin composite (Figure 2(c)).

**Figure 3:**
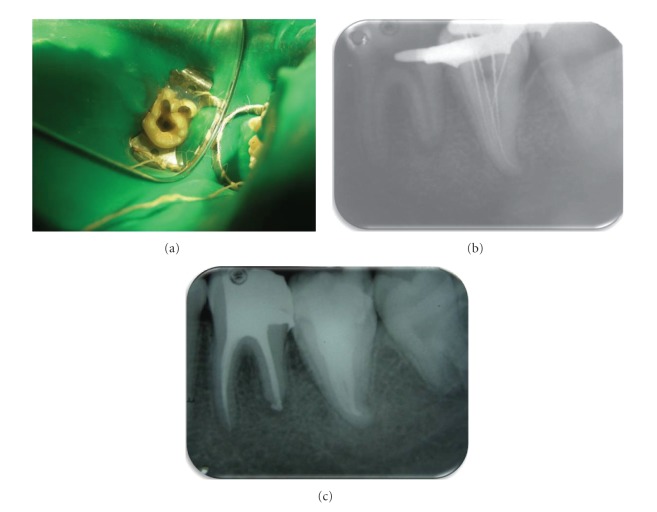
(a) Occlusal view. (b) Working length determination. (c) Postoperative final radiograph.

**Figure 4:**
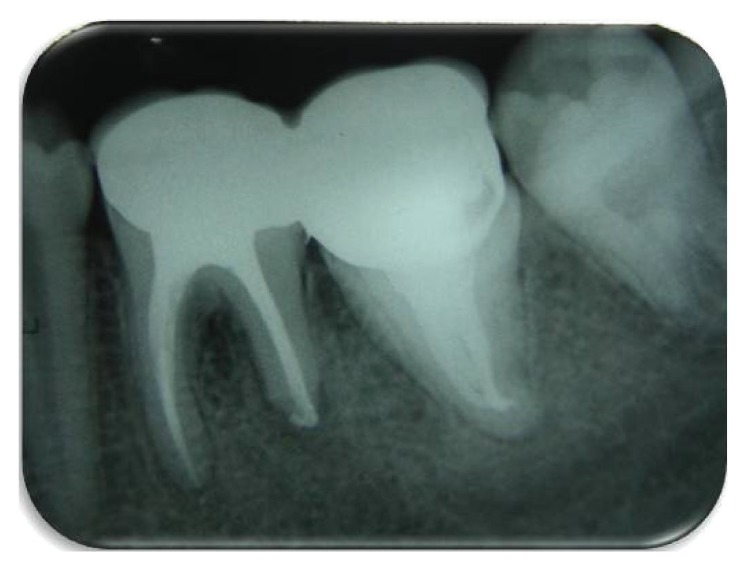
Recall radiograph 1-year postoperatively.

Routine endodontic treatment was carried out with tooth 21. The tooth was totally asymptomatic at a 1-year recall visit (Figures 3 and 4).

## 4. Discussion

Fused teeth afford a striking clinical manifestation of the differentiable and morphogenetic processes of tooth development. Fusion between supernumerary and permanent teeth occurs less frequently than fusion between other types of teeth. The incidence of unilateral occurrence is estimated in the literature to be 0.5% in the deciduous and 0.1% in the permanent dentition. The incidence of bilateral occurrence is estimated at around 0.02% for both types of dentition [1, 12, 13]. As far as the etiology for fusion is concerned, many theories have been proposed; including genetic factors, local metabolic interference during tooth bud differentiation, traumatic or inflammatory causes [1, 6, 14]. To explain fusion, some authors suggest a lack of space as the cause of deep penetration of the dental follicles. The morphology of fused teeth varies, and complex forms with separated or fused coronal pulp chambers are present. Even separated chambers can meet in the radicular area or can remain separated.

 Fusion is a developmental anomaly with inherently unusual and bizarre anatomy. When it occurs, it commonly results in caries, periodontal disease, and crowding [15]. Endodontic treatment is usually problematic, owing to the complex anatomy, tooth positioning, and difficulty in rubber dam isolation [6]. In the present case, we had to trim the prongs of the rubber dam retainer to properly seat the retainer on the tooth. A liquid dam was applied to achieve a fluid tight seal. A prerequisite for endodontic treatment of anomalous teeth is careful examination of radiographs from various angles. The amount of information gained from conventional film and digitally captured periapical radiographs is limited by the fact that the three-dimensional anatomy of the area being radiographed is compressed into a two-dimensional image. As a result of superimposition, periapical radiographs reveal limited aspects of the three-dimensional anatomy. In addition, there may also be geometric distortion of the anatomical structures being imaged [16]. These problems can be addressed by the use of SCT which can produce three-dimensional images of individual teeth and aid in better understanding of the root canal morphology.

 A new CT technique, SCT or volume acquisition CT, has been developed that has its inherent advantage. Current CT scanners have a linear array of multiple detectors, allowing multiple slices to be taken simultaneously, resulting in faster scan times and often less radiation exposure to the patient. The slices of data are then “stacked” up and can be reformatted to obtain three-dimensional images [17].

 SCT can get a large volume of data in seconds and offer more rapid examination time and with an effective dose in the range of about 1–30 mSv, which is much less than the conventional CT [18]. Christoph et al. [19] have proposed a SCT protocol with a reduced radiation dose (0.56 ± 0.06 mGy) down to the level of a single panoramic radiograph [19]. However, keeping in mind the extra cost associated, its use should be limited to cases with unusual tooth morphology.

 In this specific case, a SCT could confirm the complicated morphology of the root canal system of the fused tooth. This technique seems to have the potential to visualize the topography of root canals and offer new perspectives for dental imaging of special clinical cases.

 With the advent of newer tomographic scanners like cone beam computed tomography (CBCT) or digital volume tomography (DVT) specifically for maxillofacial and dental use, conventional scanners like SCT will be less preferred for dental imaging purposes. CBCT is advantageous in that it has a low effective dose in the same order of magnitude as conventional dental radiographs [20]. Although there has been enormous interest in the current CBCT technology, it has limitations related to the “cone-beam” projection geometry, detector sensitivity, and contrast resolution. These parameters create an inherent image “noise” that can compromise the quality of the scan and can lead to an inaccurate or false diagnosis [21]. Even though the use of CBCT involves less radiation than conventional CT, the radiation dose is still higher than regular conventional intraoral radiographs [22]. At this point of time, CBCT is limited to major metropolitan areas and is very expensive. Methods and training to improve accessibility and affordability of CBCT should be explored.

## 5. Conclusion

This paper demonstrates a predictable and successful solution towards the endodontic management of a fused mandibular second molar with a supernumerary tooth by using SCT as a diagnostic tool to a great effect in understanding the complex root canal anatomy, thus helping a great deal in rendering successful endodontic therapy. Proper diagnosis and treatment planning for endodontic management of fused teeth by using SCT can ensure predictable and successful results.
